# An Overview and Quality Assessment of European National Guidelines for Screening and Treatment of Developmental Dysplasia of the Hip

**DOI:** 10.3390/children12091177

**Published:** 2025-09-03

**Authors:** Frederike E. C. M. Mulder, Hilde W. van Kouswijk, M. Adhiambo Witlox, Nina M. C. Mathijssen, Pieter Bas de Witte

**Affiliations:** 1 Department of Orthopedic Surgery, Care and Public Health Research Institute (CAPHRI), Maastricht University, P.O. Box 5800, 6202 AZ Maastricht, The Netherlands; 2 Department of Orthopedics, Leiden University Medical Center, 2333 ZA Leiden, The Netherlands; 3 Department of Orthopedic Surgery, Care and Public Health Research Institute (CAPHRI), Maastricht University Medical Center+, 6229 AN Maastricht, The Netherlands; 4 Reinier Haga Orthopedic Center, 2725 NA Zoetermeer, The Netherlands

**Keywords:** developmental dysplasia of the hip, diagnosis, neonatal screening, therapeutics, guideline

## Abstract

**Background/Objectives**: Developmental dysplasia of the hip (DDH) is one of the most common pediatric orthopedic disorders and warrants timely diagnosis and treatment to prevent long-term disability. This review identified, summarized, and assessed the quality of current European national guidelines for DDH screening and treatment. **Methods**: Guidelines were identified by contacting the national orthopedic societies from 46 European countries and retrieving the guidelines from a recent systematic review. Two researchers independently extracted data and assessed guideline quality using the AGREE II checklist. Interrater agreement was assessed using Cohen’s κ. **Results**: Nine European national DDH guidelines were identified, of which four were published in peer-reviewed scientific journals. All guidelines advised clinical examination and imaging as part of the DDH screening program, though screening approach and timing varied considerably. Four guidelines included treatment recommendations. The type of treatment (abduction treatment vs. active monitoring) and duration of long-term follow-up showed great variation. Guideline quality ranged from 16 to 92% (Cohen’s κ = 0.62), with two out of nine guidelines rated “good quality” (>70%). **Conclusions**: European national DDH guidelines appear scarce and of varying quality and content. A coordinated European initiative is warranted to urge countries to develop evidence-based DDH guidelines using validated tools and to publish these guidelines in peer-reviewed journals, thereby advancing equal diagnosis and treatment for children with DDH.

## 1. Introduction

Developmental dysplasia of the hip (DDH) is one of the most common pediatric orthopedic disorders, with an approximated incidence of 1–3% [1]. DDH ranges from mild acetabular dysplasia with a concentrically located femoral head to severe acetabular dysplasia with complete dislocation of the hip [2]. DDH can cause pain, functional limitations, abnormal gait, and early-onset osteoarthritis [2,3]. It is estimated that DDH accounts for 9% of all hip arthroplasties and 26% of hip arthroplasties under the age of 40 years [2,4]. However, when conservative treatment is started in time, i.e., in early childhood, it is often effective [5]. Therefore, timely diagnosis and treatment are warranted to prevent disability in the long-term.

Neonatal screening programs have a preeminent role in initiating treatment at an early phase. Generally, two ultrasound (US) screening approaches exist: universal and selective screening. In a universal screening program, all infants receive US evaluation for DDH. Contrarily, in a selective screening program, US evaluations are solely performed for infants with risk factors and/or an abnormal clinical evaluation. In Europe, the timing of US evaluation ranges from week 1 to week 12 [6]. The most used US technique is according to the Graf method [7]. International screening and treatment guidelines are not available due to a lack of consensus on screening methods and timing worldwide. However, several European countries have implemented a national guideline for the screening and treatment of DDH [8,9].

To our knowledge, there is no overview of European national guidelines for screening and treatment of DDH. A consolidated overview would provide valuable insights into the current state of DDH screening across Europe and identify its shortcomings and knowledge gaps, ultimately aiming to enhance care for this vulnerable patient population. To this end, this study aimed to (1) identify the European countries that have implemented a national guideline for screening and treatment of DDH, (2) summarize the guidelines’ recommendations for screening and treatment of DDH, and (3) assess the quality of the available European national guidelines.

## 2. Materials and Methods

European orthopedic societies were identified and contacted to provide the available guideline(s) for DDH screening and treatment from their country. An email was sent to all identified societies, followed by a reminder after two weeks of non-response. Furthermore, the European guidelines reported in a recent systematic review by Krysta et al. (2024) were included in our study [9]. Non-English guidelines were translated into English by the authors, with the assistance of OpenAI ChatGPT, GPT-5, 2025 version. Two authors (FM and HK) reviewed all obtained national guidelines and extracted the relevant data according to a predetermined data extraction sheet. The following items were collected: (1) guideline: country, organization, guideline, year of publication, version, source, publication, (2) screening: clinical examination (CE), timing and place CE, factors CE, risk factors, imaging, type of imaging, imaging technique, imaging system, timing of imaging, and (3) treatment: timing of treatment, type of treatment, type of device, monitoring frequency, duration, end-point, unresolved DDH, follow-up. Using summary tables, an overview was made of the recommendations on DDH screening and treatment from the available European guidelines.

### Guideline Quality

Two authors (FM and HK) rated the quality of the guidelines according to the AGREE II (Appraisal of Guidelines for Research and Evaluation) checklist [10]. The AGREE II checklist includes 23 items organized within six domains (scope and purpose; stakeholder involvement; rigor of development; clarity of presentation; applicability; editorial independence). The AGREE II items were rated on a 7-point Likert-scale (1 strongly disagree, 7 strongly agree). Domain scores were calculated according to the following formula:

$$\mathrm{Domain}\;\mathrm{score}=\frac{\mathrm{Obtained}\;\mathrm{score}-\mathrm{Minimum}\;\mathrm{possible}\;\mathrm{score}}{\mathrm{Maximum}\;\mathrm{possible}\;\mathrm{score}-\mathrm{Minimum}\;\mathrm{possible}\;\mathrm{score}}\;*\;100\%$$

in which the minimum and maximum possible scores were determined by the number of raters [10]. For example, for a domain containing three questions, the minimum possible score with two raters would be 3 × 2 × 1 = 6. The maximum possible score with two raters would be 3 × 2 × 7 = 42.

Domain scores ranged from 0% to 100% [10]. Total scores were determined by calculating the mean of the six domain scores (range 0–100%). Total scores < 50% were rated as low, 50–70% as sufficient, and >70% as good quality, in accordance with Zhu et al. [11]. Interrater agreement for the overall checklist was assessed using the quadratically weighted Cohen’s κ [12].

## 3. Results

Of all identified European countries (n = 46, see Supplementary File S1), no orthopedic societies were identified for Andorra, Holy See, Liechtenstein, Moldova, Monaco, and San Marino. For Cyprus, an email address could not be found. The Dutch orthopedic society was not contacted since the Dutch national guideline was already known by the authors. Ultimately, 38 European orthopedic societies were contacted. In total, 7/38 European orthopedic societies responded (18%), of which 3/7 societies reported to have implemented a national guideline.

**Figure 1:**
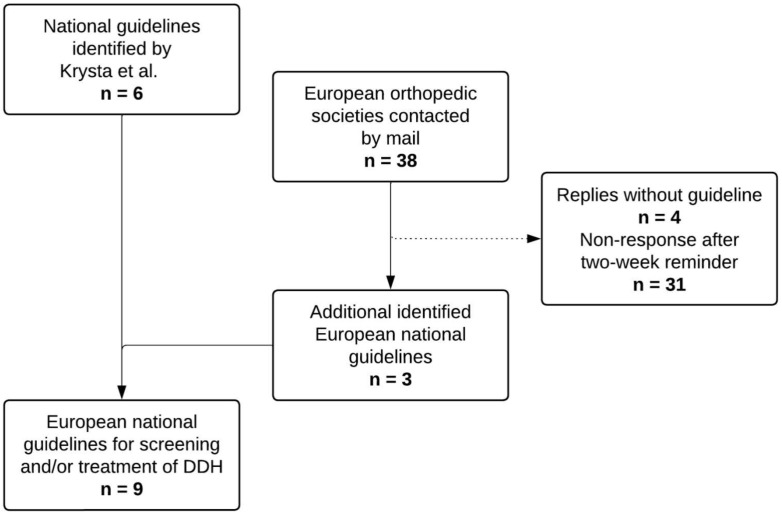
Flowchart of the identification and inclusion process of European national guidelines for DDH screening and/or treatment [9].

Combined with the results of the recent study by Krysta et al. [9], a total of nine European countries with a national DDH screening and/or treatment guideline were identified: Austria, Denmark, France, Germany, Portugal, Slovenia, Sweden, the Netherlands (NL), and the United Kingdom (UK). The flowchart of the identification process is shown in Figure 1.

All identified European national guidelines are summarized in Table 1. The oldest guideline dated from 2002 (Austria, though updated in 2013); the most recent one was updated in 2024 (UK). In total, four guidelines were published in peer-reviewed scientific journals [3,13,14,15,16].

### 3.1. Guideline Quality: AGREE II

**Figure 2:**
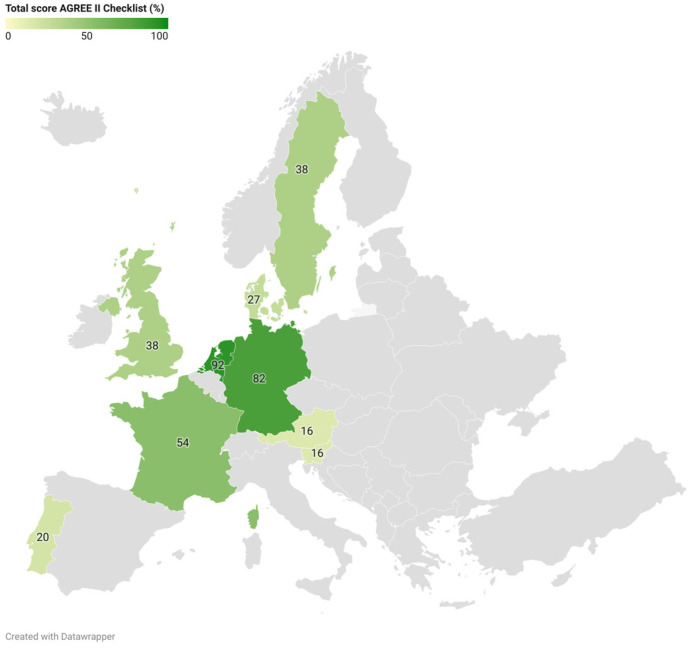
Quality of national guidelines for screening and treatment of developmental dysplasia of the hip.

The guideline quality assessment according to the AGREE II checklist is presented per guideline in Table 2 (Cohen’s κ = 0.62, 95% confidence interval 0.53–0.71). Total scores ranged from 16% (Austria, Slovenia) to 92% (NL). In total, two guidelines score as good quality (Germany, NL), one as sufficient quality (France), and six as lower quality (Austria, Denmark, Portugal, Slovenia, Sweden, and UK) (Figure 2). Overall, the domain “Scope and Purpose” shows the highest score (69%) among guidelines. The domain “Applicability” scored the lowest (20%). The year of publication did not appear to be associated with overall guideline quality, as both newer and older guidelines scored lower and higher on the checklist.

### 3.2. Guidelines for DDH Screening

#### 3.2.1. Risk Factors for DDH

In total, five guidelines report on risk factors (RF) for DDH in medical history: Denmark, France, Germany, Portugal, and NL. All five guidelines include breech presentation and familial predisposition. Other reported RF are congenital limb deformities (Denmark); other orthopedic abnormalities (France); history of oligohydramnios (Portugal); congenital foot deformities (Portugal); congenital torticollis (Portugal); polymalformative syndrome (Portugal); and asymmetric skin folds (Portugal).

#### 3.2.2. Clinical Examination (CE)

All guidelines describe CE as part of the DDH screening. CE is performed in the first week of life in most countries (Denmark, Germany, Portugal, Slovenia, Sweden, UK). In the NL, CE is performed within the first month of life, and Austria performs CE in week 4–7.

Identified relevant risk factors with CE include Barlow and/or Ortolani (Denmark, Portugal, Slovenia, Sweden, UK), limited hip abduction (Denmark, France, Germany, Portugal, Sweden, UK, NL), leg length discrepancy/Galeazzi (France, Germany, Slovenia, Sweden, UK, NL), asymmetry of skin folds (France), and deviations in walking pattern (Sweden). CE characteristics are summarized in Table 3.

#### 3.2.3. Imaging

All countries except Denmark recommend US screening of the hips. Denmark does not include US screening in the national guideline, with their screening strategies depending on regional recommendations. Most countries recommend selective US screening based on the presence of RF for DDH and/or abnormal CE (France, Portugal, Sweden, UK, NL). Austria, Germany, and Slovenia have implemented a universal screening program. The recommended timing of US screening ranges from the first week of life to the age of 3 months.

Guidelines that describe the US technique use the Graf method for identifying and classifying DDH (France, Germany, Slovenia, UK, NL). Identified imaging characteristics are summarized in Table 3.

### 3.3. Guidelines for DDH Treatment

In total, 4/9 national guidelines report recommendations for the treatment of DDH in addition to screening (Germany, Slovenia, UK, NL; Table 4).

#### 3.3.1. Centered Hips

The national guidelines of Germany, Slovenia, and the UK advise abduction treatment for infants with centered DDH. In the NL, active monitoring is advised in the first 6–12 weeks after diagnosis, followed by a Pavlik harness if no improvement or normalization has occurred after 6 or 12 weeks, respectively. With regard to abduction treatment, the German guideline recommends an orthotic device with hip flexion of 100–110° and limited hip abduction (up to 50–60°), starting within 1 week after diagnosis. The UK guideline does not elaborate on the type of harness/splint or timing of treatment. The national guideline of Slovenia recommends treatment with an Ottobock orthosis for centered DDH, preferably starting before the age of 6 weeks but no later than 3 months.

Regarding follow-up during treatment, the recommended frequency of monitoring in the guidelines ranges from every 2 weeks (UK) to every 6 weeks (NL). The endpoints of abduction treatment are normalization (alpha > 60°) in Germany, normalization in Slovenia, normalization (alpha > 60°) in the UK, and normalization or the age of 1 year in the NL.

With regard to follow-up after treatment, the national guidelines of Germany and the UK report follow-up with CE and/or X-ray at the age of 2 years, respectively. In the NL, it is advised for infants with centered DDH to have an X-ray at the age of 1, 3, and 5 years. None of the guidelines report on the follow-up of unresolved DDH after treatment of centered hips.

All information on treatment of centered DDH is summarized in Table 4.

#### 3.3.2. Decentered Hips

All national guidelines advise to start with abduction treatment for decentered hips. In addition, Germany advises closed reduction as an alternative first step in treatment. Overall, the method of abduction treatment is similar compared to centered hips in all guidelines. The UK guideline reports that treatment should be initiated no later than 2 weeks after diagnosis, whereas the timing is similar compared to centered hips for other countries.

The frequency of monitoring is not reported in the national guidelines of Slovenia and the UK. In Germany, infants with decentered hips receiving abduction treatment are advised to receive CE and US checks every 4–6 weeks. In the NL, infants with decentered hips are advised to be monitored after 3–4 weeks and 6–8 weeks of treatment. Afterwards, if the hip is centered, follow-up should occur every 6 weeks until normalization. If no improvement after 3–4 weeks or no normalization after 6–8 weeks, CR is recommended.

Treatment endpoints differ between countries and all national guidelines report treatment options for unresolved DDH (Table 4). Long-term follow-up ranges from CE and X-ray at the age of 2 years (Germany) to X-ray at the age of 1, 3, 5, 10, and 18 years (NL).

#### 3.3.3. Consolidated Recommendations Across Guidelines

To provide an overview of current recommendations across Europe, Table 5 shows the consolidated recommendations of the identified national DDH guidelines. At least three national guidelines reported the following factors with CE: Barlow, Ortolani, Galeazzi, and limited hip abduction. Also, the following risk factors for selective imaging were agreed upon by at least three guidelines: positive CE, breech presentation, family history, and other orthopedic deformities. Timing of screening through CE and/or imaging ranges from at birth to week 12. Treatment initiation (week 2 to week 12) and long-term follow-up (1 year to 5 years for centered DDH or 18 years for decentered DDH) vary greatly among national guidelines.

## 4. Discussion

This study presents an overview and quality assessment of European national guidelines for screening and treatment of DDH. In total, we have identified nine European national DDH guidelines from 46 European countries. Considering that DDH is one of the most common pediatric orthopedic disorders, this study stipulates the scarcity of European national guidelines.

The assessed guideline quality according to the AGREE II checklist differed greatly across the identified guidelines, with only two guidelines scoring as “good quality”. In particular, the items “Rigor of development”, “Applicability”, and “Editorial independence” scored poorly. It is important to recognize that some of the included national guidelines were part of broader general screening guidelines for infants and thus did not solely report on DDH. This might have impacted the AGREE II score negatively, as the focus during scoring was on the DDH chapter. However, in those cases, the complete document was screened to extract information relevant for scoring, but as the documents were not read verbatim, some details may have been missed. On the other hand, it should be emphasized that the AGREE II checklist assesses guideline quality according to reported data. Underreporting by authors negatively impacts the score, as the tool can only evaluate what is explicitly documented and may not fully reflect the true quality of the guideline. This suggests that not only are more national guidelines warranted, preferably specifically on DDH, but also that the content and reporting of the existing national guidelines should be critically reviewed. Good-quality national guidelines allow for more consistency and minimization of variations in DDH screening and treatment, thereby improving the quality of standard care [9]. The AGREE II checklist could be used as a template during guideline development to optimize guideline quality.

In total, 4/9 guidelines were published in peer-reviewed scientific journals. There appeared to be no relationship between the year of publication and guideline quality. Publication of guidelines increases findability and awareness of the existence of a guideline by its users, thereby increasing guideline adoption [17]. Therefore, we strongly recommend publishing national guidelines in peer-reviewed journals, to increase the findability of guidelines.

With regard to DDH screening, all European national guidelines advise both CE and imaging as part of the screening program. However, implementation varies greatly in terms of imaging approach (selective vs. universal) and timing of CE and/or imaging (ranging from birth to week 12). With regard to DDH treatment, only four national guidelines incorporated treatment recommendations. Similarly, large variation exists for treatment recommendations with regard to the timing of treatment initiation, monitoring frequency, treatment duration, endpoints, and long-term follow-up. Hence, this study identifies the similarities but mostly the dissimilarities in DDH screening and treatment recommendations from the scarcely available European national guidelines. We believe this variation would be even more pronounced if examined on a global scale. These dissimilarities identify knowledge gaps in current DDH care for which future research is warranted to create standardization in DDH screening and treatment. This also highlights the current input of clinician consensus on guideline development.

Based on this review we identified the following knowledge gaps and recommendations that warrant future research and awareness to improve care for children with DDH:

(1) We urge all (European) countries to develop a national guideline for DDH screening and treatment, using validated tools to ensure good guideline quality, and to publish this guideline in a peer-reviewed journal.
(2) Further studies into the (cost-)effectiveness of universal versus selective screening of DDH are warranted [18,19].
(3) Due to the large variation in the timing of screening across European countries, studies comparing initial screening moments are needed to identify the optimal age (range) for DDH screening.
(4) Studies on the (cost-)effectiveness of active monitoring versus abduction treatment for centered DDH are needed.
(5) Evaluation of the optimal timing of treatment initiation and monitoring frequency of DDH should be undertaken.
(6) Identification of the long-term follow-up moments that are needed to ensure quality care is recommended.

A strength of this study is that all European orthopedic societies were contacted to identify European national guidelines for DDH screening and treatment in addition to a literature search. Through these rigorous methods, we were able to identify national guidelines that had not been previously identified by Krysta et al. [9]. By combining these methods, we aimed to limit findability bias, since published guidelines are more accessible and discoverable. Despite sending a reminder after two weeks of not responding, there was a low response rate (18%) from the European orthopedic societies. We expect reasons for the low response rate to be related to email communication being overlooked or filtered, language barriers, non-publication of internal guidelines, or lack of coordination to the equipped expert(s). Because not all societies responded, we might have missed unpublished European national guidelines. We therefore acknowledge that the included available guidelines might not represent the full spectrum of practices across Europe. However, the guidelines included in our overview were comprehensively assessed for quality by two independent reviewers using the AGREE II checklist, adding strength to our methods. The overview was limited to European guidelines since healthcare systems, screening policies, and resource availability differ substantially between continents. This approach allows for a detailed comparison of practices within Europe. It does, however, limit the generalizability of our findings to non-European contexts. We encourage future initiatives to assess guidelines outside of Europe or on a global scale.

## 5. Conclusions

In conclusion, this study provides an overview of European national DDH guidelines. European national DDH guidelines appear scarce and of varying quality and content. Future studies should bridge knowledge gaps and achieve well-founded consensus on DDH screening and treatment to improve standard care. A coordinated European initiative is warranted to urge countries to develop evidence-based DDH guidelines using validated tools and to publish these guidelines in peer-reviewed journals. Furthermore, future studies should extend this analysis to non-European contexts to provide a global overview.

## Figures and Tables

**Table 1:** Identified European national guidelines for DDH screening and/or treatment.

Country	Organization	Guideline	Year of Publication	Source(s)	Scientific Publication(s)
Austria	Regulation of the Federal Minister for Social Security and Generations (“Verordnung des Bundesministers für soziale Sicherheit und Generationen”)	Child health check-ups under the parent–child passport program (“Eltern-Kind-Pass-Untersuchungen des Kindes”)	2002, Updated 2013	https://www.oesterreich.gv.at/themen/familie_und_partnerschaft/eltern-kind-pass/Seite.082211.html#AllgemeineInformationen ^a^	NA
Denmark	Danish Health Authority (“Sundhedsstyrelsen”)	Guidelines on preventive health services for children and adolescents (“Vejledning om forebyggende sundhedsydelser til børn og unge”)	2019, Version 3.0	https://www.sst.dk/da/udgivelser/2019/vejledning-om-forebyggende-sundhedsydelser-til-boern-og-unge ^a^	NA
France	French National Health Authority (“Haute Autorité de Santé”)	Developmental dysplasia of the hip: screening	2013	https://www.has-sante.fr/jcms/c_1680275/en/developmental-dysplasia-of-the-hip-screening ^a^	NA
Germany	German Society for Orthopaedics and Trauma Surgery (“Deutschen Gesellschaft für Orthopädie und Unfallchirurgie (DGOU)”)	S2k guideline on hip dysplasia—new registry (“S2k-Leitlinie Hüftdysplasie—neue Register-Nr.: 187-054”)	2021, Version 2.0	https://register.awmf.org/de/leitlinien/detail/187-054 ^a^	Seidl et al. (2024) [11]
Portugal	Portuguese Society of Paediatric Orthopaedics (SPOT)	Developmental dysplasia of the hip (DDH) screening protocol	Unknown	Document provided by SPOT	NA
Slovenia	NR	Developmental dysplasia of the hip −guidelines for evaluation and referral in newborns and infants in Slovenia	2019	http://www.slovenskapediatrija.si/sl-si/pdf_datoteka?revija=44&clanek=1263 ^a^	Ocepek et al. (2019) [12]
Sweden	National Board of Health and Welfare	Guidance for child health services (“Vägledning för barnhälsovården”)	2014	https://www.socialstyrelsen.se/globalassets/sharepoint-dokument/artikelkatalog/vagledning/2014-4-5.pdf ^a^	NA
United Kingdom	British Society of Children’s Orthopaedic Surgery (BSOS)	Newborn and infant physical examination (NIPE) screening programme handbook	Updated 2024	https://www.gov.uk/government/publications/newborn-and-infant-physical-examination-programme-handbook/newborn-and-infant-physical-examination-screening-programme-handbook#screening-examination-of-the-hips ^a^	Aarvold et al. (2023) [13]
The Netherlands	Dutch Orthopaedic Society (“Nederlandse Orthopedische Vereniging (NOV)”)	DDH (developmental dysplasia of the hip) in children under one year (“DDH (dysplastische heupontwikkeling) bij kinderen onder één jaar”)	2020	https://richtlijnendatabase.nl/richtlijn/ddh_dysplastische_heupontwikkeling_bij_kinderen_onder_n_jaar/startpagina_-_ddh.html ^a^	Van Bergen et al. (2022) [3] De Witte et al. (2022) [14]

NA, not applicable; NR, not reported. ^a^ Accessed on 10 June 2024.

**Table 2:** Guideline quality assessment per domain and in total using the AGREE II checklist.

Guideline Origin (Year)	Scope and Purpose (%)	Stakeholder Involvement (%)	Rigor of Development (%)	Clarity of Presentation (%)	Applicability (%)	Editorial Independence (%)	Total Score (%) ^a^
Austria (2013)	50	6	0	42	0	0	16
Denmark (2019)	56	53	8	44	0	0	27
France (2013)	89	78	34	75	19	29	54
Germany (2021)	100	89	56	92	56	100	**82**
Portugal (UNK)	50	3	3	61	2	0	20
Slovenia (2019)	25	0	3	61	4	0	16
Sweden (2014)	72	72	29	53	4	0	38
United Kingdom (2024)	75	31	27	75	10	8	38
The Netherlands (2020)	100	86	81	100	83	100	**92**
**Mean score (%)**	69	46	27	67	20	26	43

UNK, unknown. ^a^ >70%, good quality (in bold); 50–70%, sufficient quality; <50%, low quality.

**Table 3:** National guidelines for DDH screening.

Country	Clinical Examination (CE)				Imaging				
	CE	Timing and Place	CE Factors	Risk Factors	Imaging	Type	Technique	US System	Timing
Austria	Yes	Week 4–7	NR	NR	Yes	NR	NR	Universal	Week 1 and week 6–8
Denmark	Yes	After birth (midwife) and week 5 (GP)	Barlow, Ortolani, hip mobility	Breech presentation, family history (1st degree), congenital limb deformities	NR, based on regional recommendations	NR	NR	Selective	NR
France	Yes	At birth, at discharge, monthly until 3 months, each medical examination until walking age	Limited hip abduction, instability, leg length discrepancy, asymmetry of skin folds	Breech presentation, family history (1st degree), other orthopedic abnormalities	Yes	Ultrasound	Graf method	Selective	Age of 1 month when RF and/or abnormal CE
Germany	Yes	Day 3–10 (hospital/pediatric practice)	Limited hip abduction, leg length discrepancy	Breech presentation, family history	Yes	Ultrasound	Graf method	Universal	(1) Week 4–5 all newborns, ≤week 8 (2) Day 3–10 for infants with RF and/or abnormal CE
Portugal	Yes	Every consultation from birth until walking	Barlow, Ortolani, limited hip abduction	Breech presentation, family history, history of oligohydramnios, congenital foot deformities, congenital torticollis, polymalformative syndrome, asymmetric skin folds	Yes	Ultrasound	NR	Selective	(1) Week 6 when RF (2) Immediately when signs of hip instability
Slovenia	Yes	Within the first hours or days of life and every follow-up examination	Barlow, Ortolani, Galeazzi	Breech presentation, family history, syndromic anomalies, torticollis, foot deformities	Yes	Ultrasound	Graf method	Universal	(1) Week 6 all newborns (2) At maternity hospital when RF or abnormal CE NB: Graf ≥ 2A or clinically decentered hips require referral to a pediatric outpatient clinic by week 3 (but no later than month 3).
Sweden	Yes	After discharge from the maternity ward, week 4, 6 months, 10–12 months, 18 months	Barlow, Ortolani, limited hip abduction, leg length discrepancy, deviations in walking pattern	NR	Yes	NR	NR	Selective	NR
United Kingdom	Yes	At birth and week 6–8	Barlow, Ortolani, leg length discrepancy, limited hip abduction	Breech position at or after 36 completed weeks of pregnancy or at time of birth between 28 weeks and term, family history (1st degree)	Yes	Ultrasound	Graf method	Selective	(1) Within 4–6 weeks when RF and/or abnormal CE or by 40+0 weeks corrected age for babies who are born <34 + 0 weeks. (2) If screen positive results at the 6–8-week screening, then direct referral to a pediatric orthopedic surgeon and be seen by 10 weeks of age.
The Netherlands	Yes	≤month 1	Limited hip abduction (<70°), abduction difference ≥20°, Galeazzi	Breech presentation after week 32, family history	Yes	Ultrasound	Graf method	Selective	(1) 3 months when RF (2) Within 2 weeks when abnormal CE

CE, clinical examination; GP, general practitioner; RF, risk factors; NR, not reported.

**Table 4:** National guidelines for DDH treatment.

Country	Treatment									
	DDH Type	Timing	Type	Prerequisite	Device	Monitoring Frequency	Duration	Endpoint	If Unresolved	Follow-Up
Germany	Centered	<Week 1 after diagnosis	AT	NR	Orthotic device with hip flexion 100–110° and limits hip abduction to 50–60°	CE and US checks every 4–6 weeks	According to endpoint	Normalization (alpha > 60°)	NR	CE and X-ray at age 2 years
	Decentered	NR	AT or CR	NR	Orthotic device or CR followed by a hip spica cast in a squatting position	AT: CE and US every 4–6 weeks	Hip spica cast for 4–6 weeks after a successful reduction	- AT: normalization (alpha > 60°) - Hip spica cast after 4–6 weeks	- OR and hip spica cast for 4–6 weeks - Arthroscopic hip reduction and hip spica cast for 4–6 weeks	CE and X-ray at age 2 years
Slovenia	Centered	<week 6, no later than month 3	AT	Adequate hip abduction (to prevent AVN)	Ottobock orthoses	NR	Several weeks to months	Normalization	NR	NR
	Decentered	<week 6, no later than month 3	AT	Adequate hip abduction (to prevent AVN)	Ottobock orthoses	NR	Several weeks	Normalization	- Vertical traction, CR, spica cast - OR	NR
United Kingdom	Centered	NR	AT	For 2A hips: >2 weeks old	Harness/splint	CE every 2 weeks; US every 2–4 weeks	According to endpoint	Normalization (alpha > 60°)	NR	X-ray at age 2 years
	Decentered	<2 weeks after diagnosis	AT	NR	Harness/splint	NR	According to endpoint	At least 6 weeks after the hip is centered	Failing to center: Discontinue treatment for 3 weeks	X-ray at age 2 years
The Netherlands	Centered	- No improvement after 6 weeks of AM - No normalization after 12 weeks of AM	AM or AT	NR	Pavlik harness or alternative when age >6 months	CE and US every 6 weeks	According to endpoint	Normalization or age 1 year	NR	X-ray at the age of 1, 3, and 5 years
	Decentered	NR	AT	NR	Pavlik-harness or alternative when age > 6 months	- 2 times with 3–4 weeks interval. - If the hip is centered after 6–8 weeks, then every 6 weeks	- If no improvement at 3–4 weeks or not centered after 6–8 weeks, then CR - If centered after 6–8 weeks then continue AT	Normalization or age 1 year	- CR +/− adductor tenotomy followed by a spica cast for 3 months. If residual dysplasia after spica cast: Camp abduction device - If CR not successful, then OR	X-ray at the age of 1, 3, 5, 10, and 18 years

AT, abduction treatment; AM, active monitoring; AVN, avascular necrosis; CE, clinical examination; CR, closed reduction; OR, open reduction; NR, not reported; US, ultrasound.

**Table 5:** Consolidated recommendations for DDH screening and treatment across European national guidelines.

**Screening (n = 9 Guidelines)**	
**CE** First examination Follow-up examinations Factors ^a^	Yes, universal Birth—Week 7 Universal, up to the age of 18 months Barlow, Ortolani, Galeazzi, limited hip abduction
**Imaging** RF for selective imaging Modality Timing	Yes, selective (6/9) or universal (3/9) +CE, breech presentation, family history, other orthopedic deformities NR (3/9), US (6/9; Graf method (5/6)) Selective: +CE: Birth—Week 10 +RF: Week 4–Week 12 Universal: Week 1—Week 6 (depending on +CE/RF)
**Treatment (n = 4 guidelines)**	
**Centered DDH** Technique Start of treatment Follow-up (CE/US) Endpoint Long-term follow-up	Abduction treatment (3/4) or active monitoring (1/4) Week 2–Week 12 2–6 weeks interval Normalization (α-angle > 60°) Radiographs (age: varying from 1 up to 5 years)
**Decentered DDH** Technique Start of treatment Follow-up (CE/US) Endpoint Long-term follow-up	Abduction treatment (4/4) or closed reduction (1/4) Week 2–Week 12 4–6 weeks interval Normalization (α-angle > 60°) If lateralization persists: reduction (closed or open), followed by spica cast Radiographs (age: varying from 1 up to 18 years)

^a^ Mentioned in at least three of the national guidelines. +, positive; CE, clinical examination; DDH, developmental dysplasia of the hip; NR, not reported; RF, risk factors; US, ultrasound.

## Data Availability

The raw data supporting the conclusions of this article will be made available by the authors on request.

## Supplementary Materials

The following supporting information can be downloaded at https://www.mdpi.com/article/10.3390/children12091177/s1, Supplementary File S1. Identified European countries.
