# Supplementary material for: An Overview and Quality Assessment of European National Guidelines for Screening and Treatment of Developmental Dysplasia of the Hip
Source: Children (Basel). 2025 Sep 3;12(9):1177. doi: 10.3390/children12091177 (PMC12468116; doi:10.3390/children12091177)
Supplement: Supplementary file 1 [file children-12-01177-s001.zip › children-3811635-supplementary.pdf]

Supplementary File 1 – Identified European countries

1. Albania
2. Andorra
3. Austria
4. Belarus
5. Belgium
6. Bosnia and Herzegovina
7. Bulgaria
8. Croatia
9. Cyprus
10. Czech Republic
11. Denmark
12. Estonia
13. Finland
14. France
15. Germany
16. Greece
17. Holy See
18. Hungary
19. Iceland
20. Ireland
21. Italy
22. Kosovo
23. Latvia
24. Liechtenstein
25. Lithuania
26. Luxembourg
27. Malta
28. Moldova
29. Monaco
30. Montenegro
31. Netherlands
32. Norway
33. Poland
34. Portugal
35. Republic of North Macedonia
36. Romania
37. San Marino
38. Serbia
39. Slovakia
40. Slovenia
41. Spain
42. Sweden
43. Switzerland
44. Turkey
45. Ukraine
46. United Kingdom
